# Convention of Medical Colleges

**Published:** 1879-06-20

**Authors:** 


### *CONVENTION OF MEDICAL COLLEGES.*

This Body which met on the 2d of May, in Atlanta, had been appointed by the Medical College Association at its meeting in Buffalo, New York, last year, as a sort of preliminary Convention at which it was hoped that those Colleges not represented in the Medical College Association would take a part, and that a more general expression could thus be had upon the important subject of higher medical education. The result was rather a disappointment, as only twenty-three out of fifty-nine Colleges were represented, and no definite action was taken.

The College Association proper, which comprises twenty-eight Colleges, met on the 5th of May. A good deal of discussion was had, but nothing definite or satisfactory was done. Dr. Gross was elected President, and Dr. N. H. Davis, Vice-President.

### *ABINGDON ACADEMY OF MEDICINE.*

The proceedings of the above society under date of April 7th, was crowded out of the issue for which they were intended and was afterward overlooked. We now extract only a portion of the report as appropriate at this date.

This association held its annual meeting in the office of Dr. H. M. Grant, in Abingdon, Va., April 7, 1878.

Dr. J. S. Apperson, President in the Chair.

The following officers were elected for the ensuing year: Dr. W. F. Barr, President; Dr. George E. Wiley, 1st Vice-President; Dr. T. D. Kernan, 2d Vice-President; Dr. R. J. Berton, Corresponding Secretary; Dr. H. M. Grant, Recording Secretary and Treasurer.

Dr. Apperson reported a very interesting and anomalous case in practice: Abortion without pain or knowledge of the patient, no hemorrhage, complete contraction of the os on the cord, and retention of the placenta for *three months*, without producing any bad symptoms—the patient during the time remaining in good health.

The Fellows then engaged in the discussion of the subject which had been selected for the occasion, which was Rheumatism. Drs. Wiley, Apperson, Berton and Barr each gave their opinion of the disease and its treatment—which was purging, alkalies, salicylic acid, opiates and quinine, and iodide of potassium. Cupping—wet and dry—along the course of the spinal column, was highly recommended by Dr. Barr, who also remarked that he regarded colchicum as useless and worthless in *pure* Rheumatism.

Dr. Barr reported a case of Sciatica in which he had used purgatives, Quinine, wet and dry cupping and blisters over the sacrum and course of the nerve; sulphate of morphia and atropia hypodermically, with only temporary relief, but effected a cure with two hypodermic injections of chloroform into the muscle near where the nerve passes out of the pelvis. He used a syringe full each time, and inserted it *deep* into the muscle.

